# Abdominal atherosclerosis is not a risk factor of nonocclusive mesenteric ischemia among critically ill patients: a propensity matching study

**DOI:** 10.1186/s13613-022-01096-4

**Published:** 2022-12-24

**Authors:** Anhum Konan, Gael Piton, Maxime Ronot, Youness Hassoun, Hadrien Winiszewski, Guillaume Besch, Alexandre Doussot, Eric Delabrousse, Paul Calame

**Affiliations:** 1 Department of Radiology, University of Bourgogne Franche-Comté, CHRU Besançon, 25030 Besançon, France; 2 Department of Radiology, Yopougon University Hospital, 21 BP 632 Abidjan, Côte d’Ivoire; 3 Medical Intensive Care Unit, University of Bourgogne Franche-Comté, CHRU Besançon, 25030 Besançon, France; 4 Department of Radiology, University Hospitals Paris Nord Val-de-Seine, AP—HP, Beaujon, 92110 Clichy, France; 5 Surgical Intensive Care Unit, University of Bourgogne Franche-Comté, CHRU Besançon, 25030 Besançon, France; 6 Department of Digestive Surgery, University of Bourgogne Franche-Comté, CHRU Besançon, 25030 Besançon, France; 7 EA 4662 Nanomedicine Lab, Imagery and Therapeutics, University of Franche-Comté, Besançon, France; 8 Service de Radiologie, CHRU Besançon, Hôpital Jean Minjoz, 3 Boulevard Fleming, 25030 Besançon, France

**Keywords:** Atherosclerosis, Mesenteric ischemia, Nonocclusive mesenteric ischemia, Multidetector computed tomography

## Abstract

**Background:**

Although risk factors of occlusive acute mesenteric ischemia are well known, triggering factors of nonocclusive mesenteric ischemia (NOMI) remain unclear. Alongside to the known risk factors for NOMI, the role of atherosclerosis is not fully elucidated. The purpose of our study was to evaluate whether abdominal atherosclerosis is a risk factor for NOMI.

**Methods:**

From January 2018 to December 2021, all consecutive patients admitted to the intensive care unit who underwent contrast-enhanced CT for suspicion of NOMI were evaluated for inclusion. Clinical and biological data at the time of the CT scan were retrospectively extracted from medical charts and reviewed by a single radiologist. The cohorts were matched by a 1:1 propensity score based on the patient clinical, biological data, and abdominal CT features associated with NOMI. Noncontrast CT acquisitions were used to calculate calcium scores of the abdominal aorta, celiac trunk, superior mesenteric artery (SMA), and common iliac artery according to the Agatston method. Analyses were performed before and after propensity score matching.

**Results:**

Among the 165 critically ill patients included, 59 (36%) had NOMI. Before matching analysis, the SMA and total abdominal Agatston calcium scores were not different between patients without and with NOMI (52.00 [IQR = 0, 473] vs. 137.00 [IQR = 0, 259], *P* = 0.857, respectively, and 7253 [IQR = 1220, 21738] versus 5802 [IQR = 2075, 15,084]; *P* = 0.723). The results were similar after matching 38 patients with NOMI and 38 without: 153 [IQR = 0, 665] versus 85 [IQR = 0, 240] (*P* = 0.312) for the SMA calcium score, and 7915 [IQR = 1812, 21561] versus 4139 [IQR = 1440, 9858] (*P* = 0.170) for the total abdominal Agatston calcium score.

**Conclusion:**

Our results suggest that atherosclerosis is not a risk factor for NOMI in critically ill patients.

## Introduction

Acute mesenteric ischemia (AMI) is a life-threatening condition associated with a high risk of death [1, 2]. AMI is defined by the association of mesenteric vascular insufficiency (which can be occlusive or nonocclusive) with ischemic gut injury (which can be reversible or irreversible when transmural necrosis occurs). Mesenteric vessel occlusion has long been the sole cause of acute mesenteric ischemia. However, during the last decades, following the progression of care and postoperative monitoring, nonocclusive mesenteric ischemia (NOMI) has emerged [3]. NOMI is a specific etiology of AMI characterized by intestinal ischemia without arterial blood vessel occlusion [4, 5].

Risk factors of occlusive mesenteric ischemia are well known [6]: stagnant blood flow, hypercoagulability, and vascular alterations for venous OMI; heart failure, atrial fibrillation, coronary heart disease, arterial hypertension, and peripheral vascular disease for arterial OMI. Triggering factors of NOMI are not so well known. It is considered that the severity of shock per se could lead to NOMI among critically ill patients [7–9]. Hence, duration of low flow state and catecholamine infusion are established risk factors of NOMI. Two additional risk factors have been recently described [10]: enteral nutrition, which can be considered as a stress effort on the gut, and the low cardiac output and; thus, decreased oxygen delivery to the gut, highlighted by dobutamine requirement.

Alongside these risk factors, several studies have considered pre-existing abdominal atherosclerosis an evident risk factor of NOMI [11–13], favoring diminution of mesenteric flow and thus decreasing the oxygen supply to the gut. However, in-depth analysis of these studies either shows that atherosclerosis is not a risk factor in univariate analysis in the first place [8] or that it is no longer statistically significant in multivariate analysis [12, 14]. Furthermore, most of these studies focused on NOMI in the postoperative setting of cardiac surgery, whereas the etiologies of NOMI include all conditions in which a persistent low-flow state may be occurring. In addition, the assumed relationship between catecholamine infusion, vessel vasoconstriction, and NOMI is unclear. On the one hand, atherosclerosis could be considered as an additional risk factor for gut ischemia, but on the other hand, atherosclerosis may also limit the capacity of small vessels for vasocontraction. In addition, atherosclerosis could favor the development of collateral arteries from angiogenesis; thus, limiting the risk of bowel ischemia [15].

Abdominal computed tomography (CT) remains the cornerstone of NOMI diagnosis, allowing the diagnosis of bowel transmural necrosis [7] and providing prognostic elements for these patients [16]. CT also allow quantification of atheroma by detecting vascular calcifications which has been extensively studied in the coronary arteries [17, 18] by the Agatston method. However, quantification of calcification is not only possible on coronary arteries but on all arteries, and thus on arteries that supply blood to the gut [19].

Thus, we aimed to assess whether pre-existing abdominal atherosclerosis, especially when localized on mesenteric vessels, is a risk factor for nonocclusive mesenteric ischemia in critically ill patients.

## Materials and methods

### Patients

**Fig. 1:**
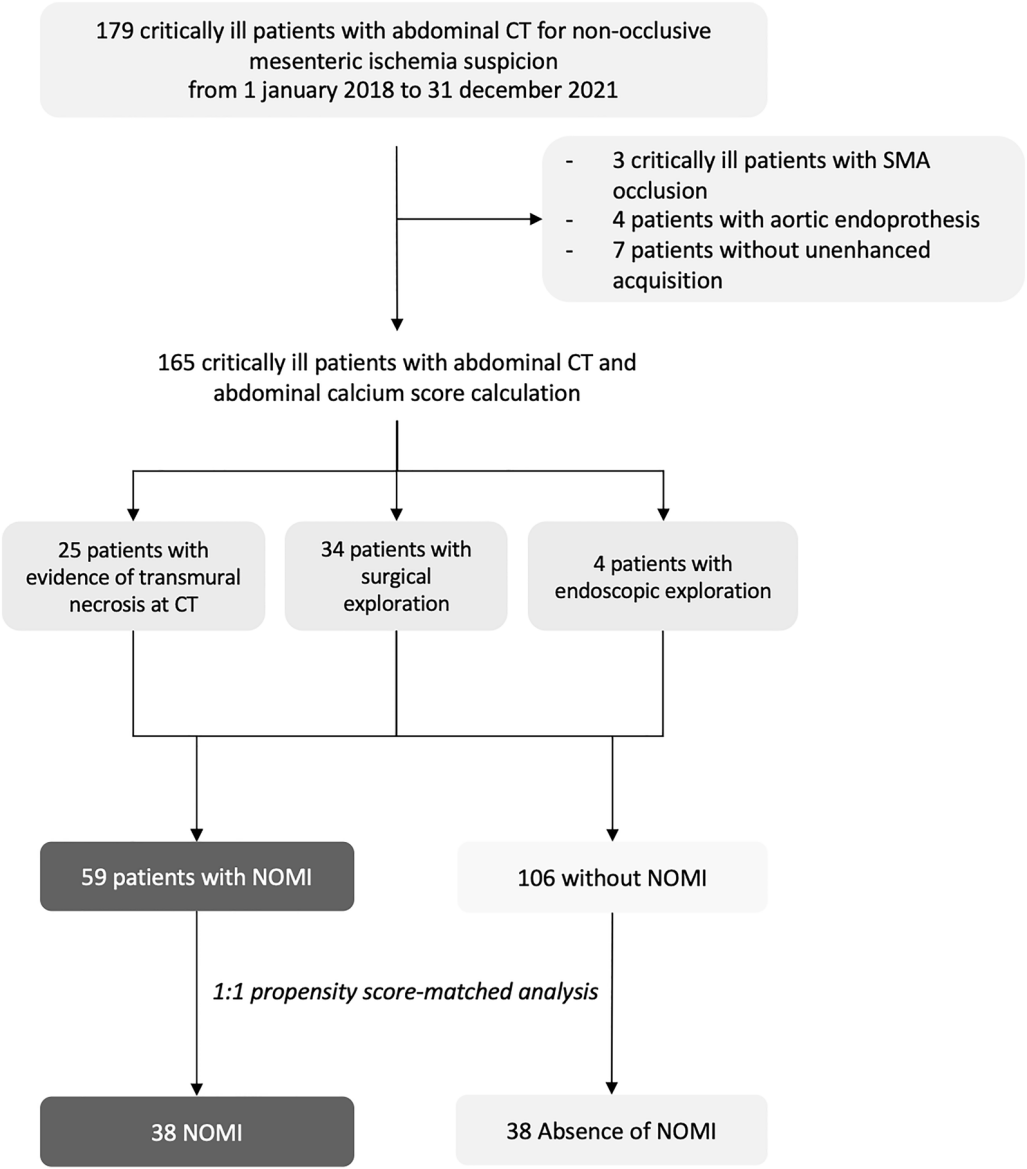
Flow chart of the study population

This was a monocentric retrospective study conducted from January 1, 2018, to December 31, 2021. All consecutive patients from ICU who underwent an abdominal CT for suspicion of NOMI were evaluated for inclusion. The inclusion criteria were: (1) critically ill patient requiring hospitalization in the ICU, (2) an available pre- and post-contrast abdominal CT. Patients with an aortic endoprosthesis and patients without available noncontrast abdominal CT were excluded because of the impossibility of computing the calcium score. Our institutional review board approved this single-center retrospective study with a waiver of informed consent. The final study population included 165 patients (Fig. 1).

### CT protocol

CT examinations were performed with a 128 MD CT scanner (Somatom Definition 128, Siemens Healthcare). In routine practice, the CT protocol for NOMI suspicion comprised noncontrast and multiphase contrast-enhanced acquisitions in the early arterial phase (acquired with bolus tracking, with a ROI located in the aorta) and portal venous phase (80 s after contrast administration). Contrast administration was performed by intravenous injection of 1.5 mL/kg of nonionic contrast medium at 400 mg I/mL through a power injector at a rate of 3–5 mL/s.

### Imaging analysis

Abdominal CT scans were blindly reviewed by a single radiologist (BLINDED) with five years of experience in the field of abdominal imaging. Noncontrast acquisitions were imported onto an AW workstation (GE Healthcare, Chicago, Illinois) to calculate the calcium scores of the abdominal aorta, celiac trunk, superior mesenteric artery (SMA), and common iliac arteries using Smartscore 4.0 software (GE Healthcare) according to the Agatston method [20]. The abdominal aorta was defined as the segment from the diaphragm to the iliac bifurcation. Ostial calcifications of the celiac trunk and the superior mesenteric artery were included in the respective arteries (celiac trunk or superior mesenteric artery).

A single radiologist blinded from the final diagnosis of NOMI measured the maximal diameter of the superior mesentery artery, celiac trunk, and inferior mesentery artery. Stenosis of the celiac trunk and superior mesenteric artery were evaluated by the NASCET method [21] using multiplanar reconstructions on a PACS workstation (Carestream Health, Rochester, NY). Vessels stenosis were considered as nonsignificant, mild, moderate and severe when they were < 25%; ≥ 25%/ < 50%; ≥ 50%/ < 70%, and ≥ 70%, respectively. Concerning the lower mesenteric artery, its small size precluded the reliable calculation of a calcium score or percentage stenosis, and thus, the diameter of its origin and the presence of stenosis or occlusion were recorded.

Reader also assessed the following CT features known to be associated with shock: spleen infarction, liver infraction, kidneys infarction, hyperenhancement of adrenal gland in the arterial phase, flattened vena cava, and diffuse splanchnic vasoconstriction.

### Endpoint measures and data collected

NOMI was defined as the evidence of bowel ischemic injury at surgical exploration or digestive endoscopy in the absence of acute mesenteric vessel obstruction. In patients without surgical or endoscopic exploration, NOMI was diagnosed when abdominal CT showed evidence of ischemic bowel injury: absence of enhancement of the bowel wall ± thinned wall for small bowel involvement [7] and absence of enhancement of the bowel wall for the colonic involvement [22].

The data were retrospectively extracted from the patients’ medical charts. Patients’ characteristics, comorbidities, and clinical data were recorded at the time of the CT. Catecholamine infusion (norepinephrine, dobutamine use) and enteral nutrition were recorded as there are known NOMI risks factors. Blood tests, blood gas were recorded at the time of the CT. The main known risk factors for NOMI are summarized in Table 1.

**Table 1:** Main risk factor of NOMI in critically ill patients according to their etiology

*Surgical*
Catecholamine infusion
Intra-aortic balloon pump support
Reoperation for bleeding
Blood product usage
Postoperative renal insufficiency
*Medical*
Catecholamine infusion
Duration of shock state
Low cardiac output
Enteral nutrition
*Cardirespiratory arrest*
Catecholamine infusion
No flow + low flow duration
Admission lactate > 5 mmol/L
Female sex
High inotropic score

### Statistical analysis

Categorical data are expressed as numbers and percentages and were compared by Pearson chi-square or Fisher’s exact test. Continuous variables are expressed as mean, standard deviation or median, interquartile range (IQR), and were compared using Student’s t-test when the distribution was normal or the Wilcoxon test when the distribution was not normal.

All calcium scores and percent stenosis are described as non-normal continuous variables. A total abdominal calcium score was calculated as the sum of the scores from the aorta, celiac trunk, mesenteric, right, and left common iliac arteries.

Patients with and without NOMI were then matched 1:1 based on their propensity scores, using the nearest neighbor method and a caliper width of 0.1 standard deviations for the propensity score. The propensity score was calculated using a multivariate logistic regression that included the main known risk factors of NOMI (noradrenaline dose and lactate levels), significant biological variables (serum pH and bicarbonate levels), and CT features of low flow state associated with NOMI (adrenal gland hyperenhancement, diffuse splanchnic vasoconstriction, and superior mesentery artery maximal diameter).

All tests were two-sided, and a *P *value < 0.05 was considered statistically significant. All analyses were performed with R version 3.4.4 (R Core Team 2017).

## Results

### Study cohort

A total of 165 critically ill patients underwent a contrast-enhanced CT for NOMI suspicion during the study period. Among them, 59 (36%) had NOMI, diagnosed by laparostomy (*n* = 30), endoscopic exploration (*n* = 4), or the sole abdominal CT (*n* = 25). The mean age of the study cohort was 68 ± 12, and there were 113/165 (68%) men. The most frequent conditions leading to the suspicion of NOMI were sepsis (64/165; 40%), followed by cardiovascular surgery (44/165; 26%) and heart failure (21/165; 13%). Seventy patients (43%) were active or past smokers, 93/165 (56%) were treated for hypertension, 57/165 (34%) had diabetes, and 46/165 (28%) had a peripheral arterial disease.

At the time of the CT, 120/165 (73%) of the patients had catecholamine infusion (median noradrenaline dose 17 mcg/min [IQR = 0; 55]. The median lactate level was 4.8 mmol/L (IQR = 2.5, 8.8), median bicarbonate level was 17 mmol/L (IQR = 13, 21), median prothrombin time was 56 (IQR = 36, 75), and median pH was 7.33 (IQR = 7.19, 7.41). Characteristics of the study population according to the presence of NOMI are provided in Table 2.

**Table 2:** Characteristics of the study population

*n*	Absence of NOMI	Presence of NOMI	*P *value
	*n* = 106	*n* = 59	
Age	68 ± 12	68 ± 12	0.747
Male sex	75 (71)	38 (64)	0.505
*Comorbidities*			
Smoking (%)	44 (42)	27 (46)	
Active	25 (24)	19 (32)	0.310
Past	19 (18)	8 (14)	0.612
Hypertension	59 (56)	34 (58)	0.936
Diabetes	29 (27)	17 (29)	0.985
Peripheral artery disease	38 (36)	19 (32)	0.763
Chronic kidney disease	16 (15)	5 (9)	0.327
Cirrhosis	17 (16)	5 (9)	0.249
Chronic congestive heart failure	15 (14)	12 (20)	0.418
Chronic obstructive pulmonary disease	17 (16)	11 (19)	0.833
Obesity (body mass index 30 kg/m2)	10 (9)	10 (17)	0.242
*Etiology of multiorgan failure*			
Cardiorespiratory arrest	9 (8)	8 (14)	0.490
Abdominal surgery	4 (4)	2 (3)	
Cardiac surgery	20 (19)	10 (17)	
Hemorrhagic shock	14 (13)	7 (12)	
Hypovolemia	3 (3)	1 (2)	
Heart failure	16 (15)	5 (8)	
Respiratory failure	1 (1)	1 (2)	
Sepsis	39 (38)	25 (42)	

Univariate analysis according to the presence of nonocclusive ischemia

*NOMI* nonocclusive mesenteric ischemia, *SD* standard deviation

Numbers in brackets are percentages

On abdominal CT, 98/165 (59%) patients had at least one organ other than the gut displaying features of ischemia (90/165 [54%], 44/165 [27%], and 36/165 [22%] for the spleen, the liver, and the kidney, respectively). Diffuse splanchnic vasoconstriction was present in 32/165 [19%] patients, Adrenal gland hyperenhancement in 130/165 [78%] patients, and flat inferior vena cava in 44/165 [27%] patients.

CT features of atherosclerosis were frequent in our study population. Only 11 (7%) patients had an abdominal calcium score of 0. The median SMA score was 83 (IQR = 0, 442; range = 0, 4652). The median total abdominal calcium score was 6690 (IQR = 0, 15,877; range = 0, 43,387). Overall, 123/165 (57%) patients had no significant SMA stenosis, 25/165 (15%) patients had mild SMA stenosis, 13/165 (8%) had moderate SMA stenosis, and 5 (3%) had severe SMA stenosis. The SMA calcium score was correlated with the degree of SMA stenosis (Pearson’s coefficient correlation 0.55 (95%CI = 0.44, 0.65; *P* < 0.001). Significant, but weak correlation was found for the celiac trunk between calcium score and degree of stenosis (Pearson coefficient correlation between 0.19 [95%CI = 0.04, 0.34; *P* = 0.014]).

The median survival was seven days (IQR = 4, 12 days), with 28-day and 90-day mortality rates of 106/165 (64%) and 119/165 (72%), respectively.

### Propensity score matching

**Fig. 2:**
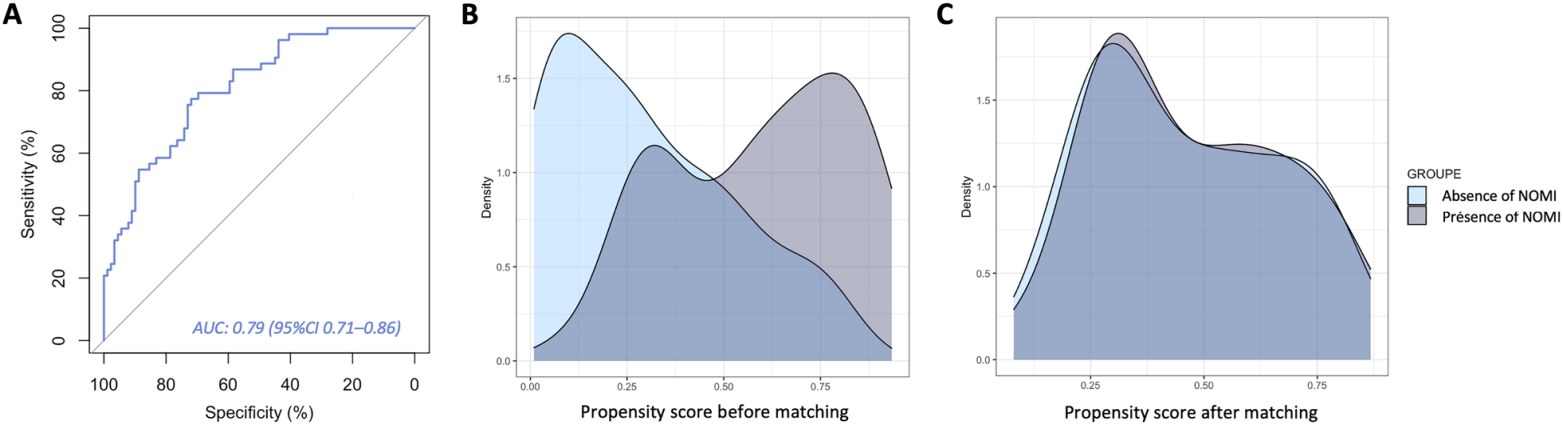
**A** ROC curve of the propensity score for nonocclusive mesenteric ischemia prediction. **B** Density plot shows the propensity score distribution before propensity score matching analysis. **C** Density plot shows the propensity score distribution after propensity score matching analysis

The standardized mean differences (SMD) of all clinical and biological variables input into the multivariate model were < 0.20 after propensity score matching (i.e., noradrenaline dose, lactate level, bicarbonate level, pH) (Table 3). All SMD of adjusted CT variables input into the multivariate model were also < 0.20 after PSM (propensity score matching) (i.e., presence of diffuse vasosplanchnic vasoconstriction, adrenal gland hyperenhancement, flat inferior vena cava, liver infarction, kidney infarction, spleen infarction, and superior mesenteric artery diameter). The area under the ROC curve (AUROC) of the propensity score for the prediction of NOMI was 0.79 (95%CI 0.71–0.86). Performances and density plots of the propensity score before and after propensity score are presented in Fig. 2.

**Table 3:** Characteristics of the study population at the time of the CT before and after propensity score matching

*n*	Before propensity score matching		*P *value	SMD	After propensity score matching		*P* value	SMD
	Absence of NOMI	Presence of NOMI			Absence of NOMI	Presence of NOMI		
	*n* = 106	*n* = 59			*n* = 38	*n* = 38		
*Clinical and biological variables*								
catecholamine infusion	23 (23)	15 (25.9)	0.832	0.340	28 (77.8)	24 (66.7)	0.302	0.250
Norepinephrine dose (mcg/min)	17 [5, 37]	27 [3.3, 67]	**0.195**	0.351	21 [2.5, 52]	18 [0, 50]	0.636	0.079
Dobutamine	15 (16.7)	10 (15.2)	0.973	0.098	7 (20.0)	5 (13.5)	1.000	0.174
Enteral nutrition	14 (13)	8 (0.14)	0.926	0.015	0.08 (0.28)	0.14 (0.35)	0.486	0.164
ASAT, UI/L	117 [44, 545]	102 [53, 715]	0.791	0.102	121 [54, 727]	108 [52, 650]	0.958	0.182
Total bilirubin (mmol/l)	19 [10, 42]	17 [10, 42]	0.794	0.111	19 [11, 44]	13 [9.5, 40]	0.356	0.368
PT (%)	56 ± 25	54 ± 29	0.564	0.094	58 ± 27	61 ± 30	0.653	0.107
CRP (mg/L)	116 [37, 246]	85.40 [41.5, 171]	0.717	0.263	63 [21, 137]	99 [42, 147]	0.409	0.200
pH	7.31 ± 0.16	7.25 ± 0.17	**0.027**	0.360	7.29 ± 0.2	7.30 ± 0.2	0.827	0.050
PaO_2_, mmHg	12.74 ± 8.90	14.4 ± 9.77	0.271	0.177	12.10 ± 7.35	14.05 ± 10.24	0.343	0.219
PaCO_2_, mmHg	4.65 ± 1.82	4.84 ± 1.97	0.528	0.101	5.09 ± 2.45	4.92 ± 2.23	0.748	0.074
Bicarbonates, mmol/l	17.51 ± 5.77	15.79 ± 5.81	**0.069**	0.297	17.53 ± 5.50	17.26 ± 5.90	0.838	0.047
Lactates, mmol/l	5.73 ± 4.68	6.80 ± 4.60	**0.160**	0.230	5.34 ± 4.30	6.07 ± 4.33	0.467	0.168
Platelets, (G/l)	139 [82.50, 225.50]	148 [79, 245]	0.801	0.044	143 [92, 213]	160 [106, 257]	0.380	0.264
Hemoglobin, g/dl	9.87 ± 2.47)	9.74 ± 2.50	0.741	0.054	9.95 ± 2.66	9.84 ± 2.60	0.852	0.043
*CT variables*								
Diffuse splanchnic vasocontriction	11 (10.5)	21 (36.8)	** < 0.001**	0.653	7 (18.4)	10 (26.3)	1.000	< 0.001
Adrenal gland hyperenhancement	74 (69.8)	56 (94.9)	** < 0.001**	0.698	36 (100.0)	34 (94.4)	0.619	0.115
Flattened vena cava	22 (20.8)	22 (37.3)	**0.034**	0.368	0.32 (0.47)	0.26 (0.45)	1.000	0.056
Liver infarction	18 (17.0)	26 (44.1)	** < 0.001**	0.615	12 (31.6)	13 (34.2)	1.000	0.056
Kidney infarction	13 (12.3)	23 (39.0)	** < 0.001**	0.643	6 (15.8)	9 (23.7)	0.562	0.199
Spleen infarction	47 (44.8)	43 (72.9)	** < 0.001**	0.613	26 (68.4)	26 (68.4)	1.000	< 0.001
Superior mesenteric artery diameter (mm)	5.99 ± 1.36	5.34 ± 1.53	**0.005**	0.451	5.65 1.41	5.63 ± 1.53	0.954	0.083
Coeliac trunk diameter (mm)	5.79 ± 1.75	5.11 ± 2.07	**0.007**	0.352	5.8 ± 1.8	5.49 ± 2.17	0.167	0.173
Inferior mesentery artery (mm)	2.3 [1.8, 2.8]	1.65 [1.00, 2.40]	**0.004**	0.224	2.05 [1.80, 2.50]	1.80 [1.07, 2.88]	0.156	0.044

Numbers in brackets are percentages for qualitative variables

Non-normal quantitative variables are expressed as median and interquartile range and compared using Wilcoxon-Test

Normal quantitative variables are expressed as mean ± standard deviation and compared using student test

Bold value are variables included in the propensity matched analysis

*CT* computerized tomography, *NOMI* nonocclusive mesenteric ischemia, *ASAT* aspartate aminotransferase, *PaCO*_*2*_ arterial blood carbon dioxide tension, *PaO*_*2*_ arterial blood oxygen tension, *SD* standard deviation, *PT* prothrombin rate, *SMD* Standard mean deviation

### Atherosclerosis and NOMI

**Fig. 3:**
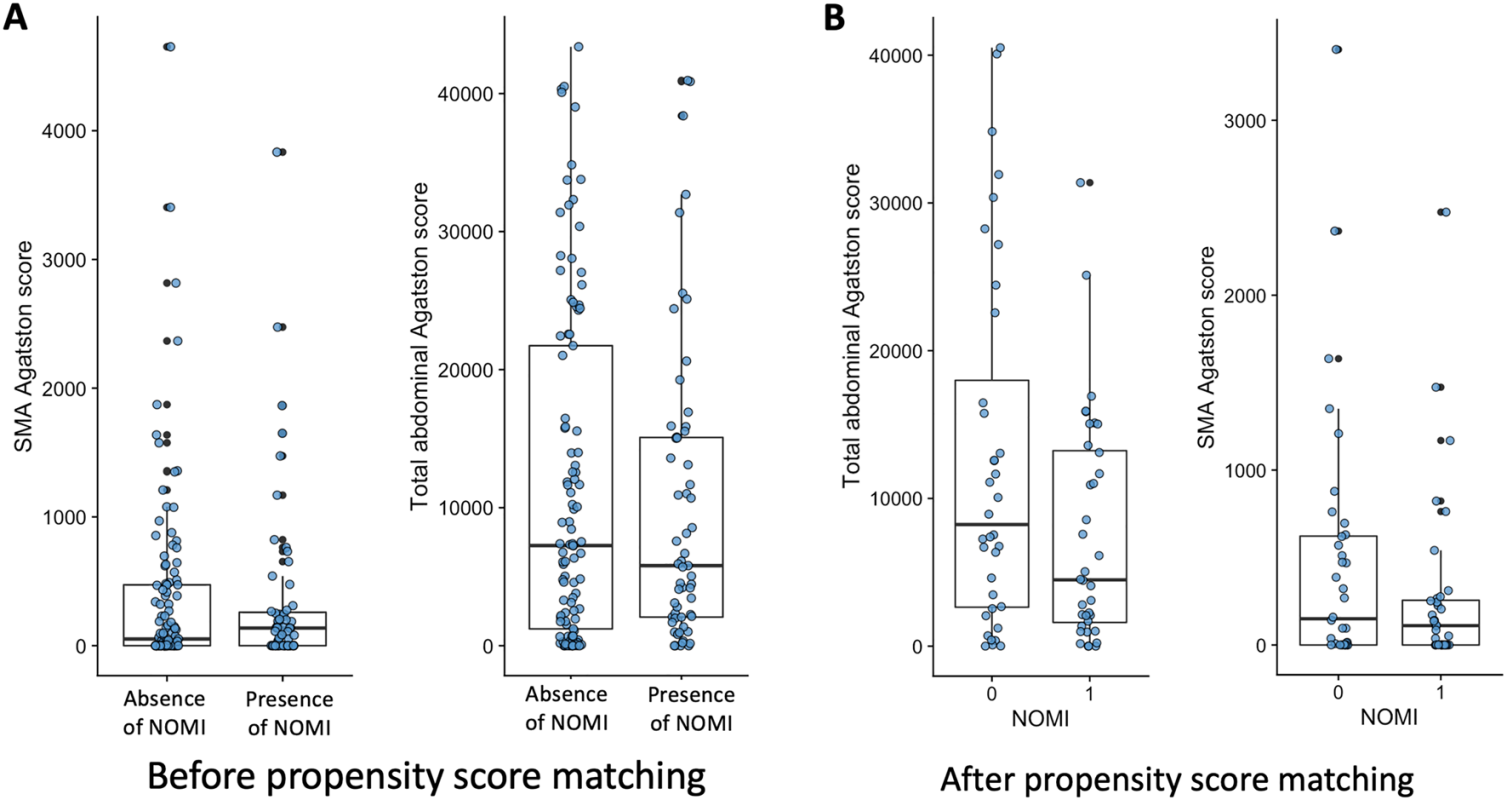
Boxplots of the distribution (median and interquartile range) of superior mesenteric artery and total abdominal calcium score according to nonocclusive mesenteric ischemia. Lines are median and interquartile range. **A** Before propensity score matching analysis. **B** After propensity score matching analysis. *SMA* Superior mesenteric artery

Before PSM, the 59 patients with confirmed NOMI showed no difference in overall atherosclerosis burden compared with those without NOMI (Table 4). The calcium score of the SMA was not different between groups (median 52 [IQR = 0, 473] in patients without NOMI vs. 137 [IQR = 0, 259] in patients with NOMI, *P* = 0.857 (Fig. 3). The percentage of SMA stenosis was not different between patients without or with NOMI (median of 0 [IQR = 0, 31] vs. 0 [IQR = 0, 18] respectively; *P* = 0.259). The calcium score of the celiac trunk was not different between patients without and with NOMI (median 49 [IQR = 0, 312] vs. 137 [IQR = 0, 275] respectively; *P* = 0.853). The percentage of celiac trunk stenosis was not different between patients without or NOMI (median 16 [IQR = 0, 41] vs. 26 [IQR = 0, 47] respectively; *P* = 0.495).

**Table 4:** Atherosclerosis-related CT variables before and after propensity score matching

*n*	Before propensity score matching		*P *value	After propensity score matching		*P *value
	Absence of NOMI	Presence of NOMI		Absence of NOMI	Presence of NOMI	
	*n* = 106	*n* = 59		*n* = 38	*n* = 38	
*Atherosclerosis-related variables*						
Coeliac trunk						
Calcium score	49 [0, 312]	79 [0, 275]	0.853	99.50 [0, 286]	22 [0, 238]	0.300
Nascet % stenosis	16 [0, 41]	26 [0, 47]	0.495	10 [0, 44]	25 [0, 46]	0.885
None	38 (36.5)	21 (35.6)	0.954	13 (34.2)	15 (39.5)	0.796
< 25%	18 (17.3)	8 (13.6)		7 (18.4)	5 (13.2)	
> 25%/< 50%	31 (29.8)	19 (32.2)		14 (36.8)	11 (28.9)	
> 50%/< 70%	12 (11.5)	7 (11.9)		3 (7.9)	5 (13.2)	
≥ 70%	5 (4.8)	4 (6.8)		1 (2.6)	2 (5.3)	
Superior mesentery artery						
Calcium score	52.00 [0, 473]	137 [0, 259]	0.857	153 [0, 665]	85 [0, 240]	0.312
Nascet % stenosis	0 [0, 31]	0 [0, 0.18]	0.259	0 [0, 32]	0 [0, 4]	0.113
None	56 (53.3)	38 (64.4)	0.281	20 (54.1)	28 (73.7)	0.206
< 25%	18 (17.1)	9 (15.3)		6 (16.2)	5 (13.2)	
> 25%/ < 50%	20 (19.0)	5 (8.5)		7 (18.9)	1 (2.6)	
> 50%/ < 70%	9 (8.6)	4 (6.8)		3 (8.1)	3 (7.9)	
≥ 70%	2 (1.9)	3 (5.1)		1 (2.7)	1 (2.6)	
Inferior mesentery artery						
Stenosis (%)	23 (21.7)	10 (16.9)	0.592	9 (23.7)	6 (15.8)	0.564
Obstruction	11 (10.3)	11 (18.6)	0.209	4 (10.5)	6 (15.8)	0.734
Aortic calcium score	4248 [789, 12900]	4893 [1132, 9988]	0.837	4406 [1130, 15348]	2375 [993, 5950]	0.226
Right iliac artery calcium score	846 [155, 2758]	697 [151, 2281]	0.571	1331 [145, 2855]	384 [86, 959]	0.127
Left iliac artery calcium score	860 [73, 2413]	576.00 [209, 2191]	0.851	995 [89, 2006]	441.50 [75, 861]	0.116
Total abdominal calcium score	7253 [1220, 21738]	5802 [2076, 15085]	0.723	7916.50 [1812, 21561]	4139 [1439, 9858]	0.170

Numbers in brackets are percentages for qualitative variables

Non-normal quantitative variables are expressed as median and interquartile range and compared using Wilcoxon test

*CT* computerized tomography, *NOMI* nonocclusive mesenteric ischemia

After PSM, 38 pairs of patients were retained for analysis. In the matched analysis, atherosclerosis variables were not different between patients with and without NOMI (Table 4). The SMA calcium score was a median of 153 [IQR = 0, 665] in patients without NOMI vs. 85 [IQR = 0, 240] in a patient with NOMI (*P* = 0.312). The median SMA stenosis was not different in patients without or with NOMI (median 0% [IQR = 0, 34] vs. 0% [IQR = 0, 11], respectively; *P* = 0.083). The total abdominal calcium score was not different between patients without or with NOMI (median 7917 [IQR = 1812, 21561] vs. 4139 [IQR = 1440, 9858], respectively; *P* = 0.170). Abnormalities (i.e., stenosis or occlusion) of the inferior mesentery artery were not different between patients without or with NOMI.

## Discussion

This study aimed to assess the relationship between pre-existing abdominal atherosclerosis and NOMI in critically ill patients. After propensity score matching analysis according to the main known risk factors of NOMI and CT features of low flow state, we showed that abdominal atherosclerosis, especially when localized on mesenteric vessels, is not a risk factor for NOMI.

The relationship between NOMI and abdominal atherosclerosis is challenging to elucidate. Previous studies evaluated only “peripheral atherosclerosis disease” [9, 13, 14] as an atherosclerosis marker. Even if this marker was associated with the development of NOMI, mainly in the context of cardiac surgery, it does not qualify atherosclerosis as a risk factor for NOMI but may reflect confusion biases. Indeed, the postoperative course after cardiac surgery may be more complicated in a patient with pre-existing atherosclerosis, with no role for atherosclerosis per se in developing NOMI. For example, Nilsson et al. [12] showed that peripheral arterial disease was a risk factor for NOMI after cardiac surgery in univariate analysis (present in 2388/18862 (13%) vs. 7/17 (41%) of patients without vs. patients with NOMI; *P* < 0.001). However, the multivariate analysis identified only postoperative features as being significantly associated with NOMI and excluded atherosclerosis.

We specifically evaluated atherosclerosis of the mesenteric vessels. Furthermore, as the calcium score imperfectly characterizes the atherosclerotic disease burden, we also evaluated other parameters such as stenosis of the mesenteric vessels. None were found associated with NOMI. Indeed, after PSM analysis of critically ill patients with comparable levels of visceral failure, low flow, and catecholamine doses, we observed similar atherosclerosis burden, by calcium score, but also percentage of mesenteric vessels stenosis evaluation. Further studies should now focus on the prognostic value of atherosclerosis in critically ill patients. Although atherosclerosis is a risk factor for poor outcomes in patients with NOMI [19], it does not seem to play a specific role in the occurrence of NOMI. Therefore, NOMI should be suspected in view of the severity of shock and low-flow states, high dose of catecholamines, marked lactic acidosis, increased plasma creatinine, and hepatic failure more than pre-existing cardiovascular risk factors or atheromatous burden.

Other factors are associated with NOMI. First, we found that all CT features belonging to the hypoperfusion complex [19, 20, 23], i.e., the hollow adrenal gland sign [24], organ infarction, flattened inferior vena cava, vasoconstriction of mesenteric vessels [5, 25–27] were all associated with NOMI. Indeed, NOMI is the ultimate consequence of prolonged gut hypoperfusion and has a poor prognosis. Abdominal CT has a major role as an initial diagnostic modality in abdominal emergencies [28–30], but also in critically ill patients [7, 22] by detecting bowel ischemia and necrosis. Further studies will now have to focus on earlier steps of gastro-intestinal failure such as such as a potential correlation between CT features and biomarkers of endothelial dysfunction [31], but also with biomarkers of mucosal gut damage [32], and their association with intra-abdominal pressure. To this end, in assessing changes in microcirculation along the gut in critically ill patients, dual-energy CT will certainly have a role to play in quantifying the change in vascularity by iodinate concentration measurement.

Our study has limitations. First, this retrospective study is subject to a selection bias. Indeed, we included only patients with NOMI suspicion and an available enhanced abdominal which have selected a very poor prognosis cohort that suffer from multiorgan failure, with high prevalence of atherosclerosis. Although performance of propensity score was good, matching analysis on other severity scores could have strengthened PSM. Then, a relatively high proportion of our cohort (i.e., 24/59; 41%) did not have surgical or endoscopic confirmation of NOMI due to their poor prognosis. However, our group previously identified reliable CT findings of NOMI [7, 18], and we believe that the risk of underdiagnosis of NOMI remains low (Specificity of absence of enhancement range from 79 to 97% according to the bowel segment involved [22]). Because of the time required by the calculation of abdominal calcium scores, the number of patients in the study is relatively limited. Thus, prevalence of significant mesenteric vessels stenosis is low, and study may be underpowered. Finally, morphologic CT evaluation may not reflect the hemodynamic change induce by vessels stenosis.

In conclusion, our results suggest that the calcium score and stenosis of the three main mesenteric arteries are not associated with an increased risk of NOMI. The main factors associated with NOMI are related to the severity and the duration of shock but are not related to the abdominal atherosclerosis burden.

## Data Availability

The datasets used and analyzed during the current study are available from the corresponding author on reasonable request.

## Abbreviations

AMI: Acute mesenteric ischemia
ASAT: Aspartate aminotransferases
IQR: Interquartile range
NOMI: Nonocclusive mesenteric ischemia
OMI: Occlusive mesenteric ischemia
CT: Computed tomography
PACS: Picture archiving and communication system
SMA: Superior mesenteric artery
PSM: Propensity score matching
SMD: Standardized mean difference
ICU: Intensive care unit
